# Assessment of Corneal Pachymetry Distribution and Morphologic Changes in Subclinical Keratoconus with Normal Biomechanics

**DOI:** 10.1155/2019/1748579

**Published:** 2019-11-19

**Authors:** Peng Song, Kaili Yang, Pei Li, Yu Liu, Dengfeng Liang, Shengwei Ren, Qingyan Zeng

**Affiliations:** ^1^Aier School of Ophthalmology, Central South University, Changsha, China; ^2^Hankou Aier Eye Hospital, Wuhan, China; ^3^Wuhan Aier Eye Institute, Wuhan, China; ^4^Henan Provincial People's Hospital, Henan Eye Hospital, Henan Eye Institute, People's Hospital of Zhengzhou University, Zhengzhou, China

## Abstract

**Purpose:**

To investigate the pachymetry distribution of central cornea and morphologic changes in subclinical keratoconus with normal biomechanics and determine their potential benefit for the screening of very early keratoconus.

**Methods:**

This retrospective comparative study was performed in 33 clinically unaffected eyes with normal topography and biomechanics from 33 keratoconus patients with very asymmetric ectasia (VAE-NTB; Corvis Biomechanical Index defined) and 70 truly normal eyes from 70 age-matched subjects. Corneal topographic, tomographic, and biomechanical metrics were measured using Pentacam and Corvis ST. The distance and pachymetry difference between the corneal thinnest point and the apex were defined as D_TCP-Apex_ and DP_TCP-Apex_, respectively, to evaluate the pachymetry distribution within the central cornea. The discriminatory power of metrics was analysed via the receiver operating characteristic curve. A logistic regression analysis was used to establish predictive models.

**Results:**

The parameters, D_TCP-Apex_ and DP_TCP-Apex_, were significantly higher in VAE-NTB than those in normal eyes. For differentiating normal and VAE-NTB eyes, the Belin-Ambrósio deviation (BAD-D) showed the largest area under the curve (AUC; 0.799), followed by ARTmax (0.798), D_TCP-Apex_ (0.771), tomography and biomechanical index (0.760), maximum pachymetry progression index (PPImax, 0.756), DP_TCP-Apex_ (0.753), and back eccentricity (B_Ecc, 0.707) with no statistically significant differences among these AUCs. In the VAE-NTB group, the parameter B_Ecc was significantly and positively correlated with D_TCP-Apex_ (*P*=0.011) and DP_TCP-Apex_ (*P*=0.035), whereas the posterior elevation difference had a significant positive association with DP_TCP-Apex_ (*P*=0.042). A model using the indices D_TCP-Apex_, B_Ecc, PPImax, and index of height asymmetry demonstrated the highest AUC of 0.846 with 91.43% specificity.

**Conclusions:**

Abnormal pachymetry distribution within the central cornea and subtle morphologic changes are detectable in subclinical keratoconus with normal biomechanics. This may improve VAE-NTB eyes detection.

## 1. Introduction

The early detection of subclinical keratoconus (KC) is imperative to promptly choose between refractive surgery and alternative treatment options [1, 2]. Scheimpflug tomography facilitates the accurate evaluation of regional corneal pachymetry and elevation; additionally, it has been considered the best available diagnostic modality for early keratoconus (KC) [3]. Published studies indicated that the central corneal thickness was a useful parameter to identify clinical KC and evaluate KC progression [4, 5]. Furthermore, the corneal thickness spatial profile and percentage thickness increase, along with the pachymetry progression indices (PPI), provided a comprehensive understanding of the thickness of the entire cornea. Moreover, these parameters had been proven to have high accuracy for diagnosis of clinical KC [6, 7]. Although KC populations have significantly thinner corneas than healthy individuals, we still need to intensively discuss whether the central corneal thickness alone is suitable for the diagnosis of subclinical KC. Additional considerations should be taken into account, including the following: (1) the thickness values of thin non-KC corneas partially overlap with the subclinical KC corneas [8, 9], and (2) corneal thickness differs among eyes from different regions and ethnicities [10]. For instance, Chan et al. [11] reported that the best cutoff value for the Ambrósio relational thickness to differentiate subclinical KC from normal eyes was 386.5, but a similar mean value of 381.8 was determined in thin non-KC corneas by Huseynli et al. [8]. Therefore, not all predictors for clinical KC may be considered appropriate to screen subclinical KC.

Topical KC changes, including corneal thinning, elevation, and steeping, result from a vicious cycle of corneal stroma loss and biomechanical failure [12, 13]. Ultrastructural analyses revealed that KC involved altered lamellar arrangements and abnormal intralamellar cohesion [14], which may be responsible for early KC signs. Theoretically, silent corneal thinning and biomechanical instability occur in the weakest area of the cornea at the onset of KC; however, topographic abnormalities are rarely identified. During KC development, the pathophysiological defect of keratoconic cornea undergoes transformations from quantitative to qualitative changes. Studies have demonstrated that biomechanical parameters enable accurate screening for subclinical KC eyes, even for those with normal topography [15, 16]. The overall biomechanical resistance of the cornea was determined, to a varying extent, using the corneal thickness (or corneal volume), internal structure, and extracellular matrix; any factor that changes the structure of the cornea may influence the biomechanical properties of the same [17]. At the very early stage of KC, the corneal entities continue to have similar thickness to normal eyes and may provide adequate biomechanical resistance to maintain a normal corneal shape. Furthermore, the biomechanical instability caused by subtle changes to the corneal infrastructure at the onset of KC may not be detectable in an in vivo measurement. However, the first detectable signs of subclinical KC, regardless of either tomographic or biomechanical abnormalities, remain uncertain [11, 18].

The locations and relative pachymetry of corneal reference points reflect corneal properties and are extremely useful to evaluate the status of corneal ectasia [19]. The identification of central cornea alterations in biomechanically normal subclinical KC may promote further insight into early KC development and optimize subclinical KC screening. Therefore, this study aimed to investigate the pachymetry distribution in the central corneal area of very early subclinical KC with normal biomechanics and determine the potential benefit of early KC screening.

## 2. Methods

This retrospective comparative study adhered to the tenets of the Declaration of Helsinki and was approved by the Institutional Review Board of Hankou Aier Eye Hospital (Wuhan, China). Written informed consent was obtained from each participant.

All participants underwent a comprehensive ophthalmic examination by an experienced anterior segment expert, including subjective analysis using slit-lamp biomicroscopy, and objective examinations such as Corvis ST and Pentacam HR examinations with acceptable quality for accurate analysis. All enrolled patients had clear evidence of KC in one eye and no clear evidence of disease in the clinically unaffected eye. The clinically keratoconic eyes had a topographical keratoconus classification (TKC) value of 1 or greater, focal corneal thinning, anterior/posterior steepening, visual blurring and/or distortion, scissoring on retinoscopy, and at least one of the slit-lamp findings, such as stromal thinning, Fleischer ring, Vogt's striae, or Munson's sign. The diagnosis of clinically keratoconic eyes was used to identify unilateral KC patients, but not for further analysis in this study.

Age-matched individuals with normal eyes were recruited from a population of healthy participants who had undergone uneventful refractive surgery and had no postoperative corneal ectasia for two years. These normal individuals of the control group presented the following characteristics in both eyes: normal clinical evaluation, corrected distance acuity of 20/20 or better, normal Scheimpflug imaging (TKC = 0), and a Corvis Biomechanical Index (CBI) of <0.3. In this study, we considered a CBI <0.3 as an indicator of normal biomechanics. Only one eye of every normal participant was randomly selected for further statistical analyses. The clinically unaffected eyes of very asymmetric KC patients that met the inclusion criteria for the control group were defined as very asymmetric ectasia eyes with normal biomechanics (VAE-NTB).

The following criteria were applied to all eyes included in the study: no contact lenses worn for at least 2 (soft contact lenses) or 4 (rigid contact lenses) weeks prior to examination, no history of eye diseases, no previous ocular surgery, and no use of topical eye medication besides artificial tears. Finally, the study included 70 eyes in the control group and 33 eyes in the VAE-NTB group.

The techniques for Pentacam and Corvis ST analyses have been previously described [20]. All measurements with the Corvis ST and Pentacam HR (both Oculus Optikgeräte GmbH) were performed by experienced technicians. Further calculations and analyses were performed only when the “QS” buttons of the Pentacam HR and Corvis ST read “OK.” The corneal thickness, sagittal curvature, Belin/Ambrósio enhanced ectasia, and CBI display maps were evaluated. The following indices provided by Scheimpflug and Corvis ST imaging were recorded: corneal apex thickness, minimum corneal thickness, the coordinates of the corneal thinnest point, mean keratometry (Km), astigmatism, eccentricity (Ecc), maximum keratometry (Kmax), PPI, ARTave, ARTmax, index of surface variation (ISV), index of vertical asymmetry (IVA), index of height asymmetry (IHA), index height decentration (IHD), keratoconus index (KI), central keratoconus index (CKI), Belin-Ambrósio deviation (BAD-D), posterior corneal elevation difference (B_Elv-D), Ambrósio's relational thickness to the horizontal profile (ARTh), CBI, and tomography and biomechanical index (TBI) values. The distance in the *XY* plane and the pachymetry difference between the corneal thinnest point and the apex were defined as D_TCP-Apex_ and DP_TCP-Apex_, respectively.

## 3. Statistical Analysis

Statistical analyses were performed using statistical package for the social sciences (SPSS) 23.0 software and MedCalc software. Descriptive results are presented as mean ± standard deviation or the median (M_25_, M_75_). All data were analysed with the Kolmogorov-Smirnov normality test and Levene's test for equal variances to choose the appropriate method, and differences between the two groups were compared using the independent *t*-test or Mann–Whitney *U* test. Bivariate normal analyses were conducted before the Pearson or Spearman correlation tests to determine the association between variables. The receiver operating characteristic (ROC) curve was used to test the discriminatory power of studied metrics in differentiating the study populations. A multivariable logistic regression analysis was used to establish a combined model. Pairwise comparisons of the area under the ROC curve (AUC) were accomplished with the nonparametric Delong test. With regard to each multivariable analysis, Wald's chi-squared test was used to remove the least influential variable in a stepwise manner to maximize the AUC values using a minimal number of variables. Two-tailed *P* values <0.05 were considered statistically significant.

## 4. Results

The study included 70 eyes in the control group and 33 eyes in the VAE-NTB group with mean ages of 24.7 ± 5.2 and 24.1 ± 5.6 years, respectively, and no statistically significant difference was found between the two groups (*P*=0.779). The descriptive values and results of the ROC curve analysis comparing the normal with the VAE-NTB eyes are shown in Table 1. Comparison of the two groups revealed statistically significant differences in the following parameters: back Ecc (B_Ecc), D_TCP-Apex_, DP_TCP-Apex_, mean PPI (PPImean), maximum PPI (PPImax), ARTave, ARTmax, ISV, IVA, IHA, IHD, KI, B_Elv-D, BAD-D, and TBI; however, no significant differences were found in other parameters including front Km, front astigmatism, front Ecc, back Km, back astigmatism, Kmax, minimum PPI (PPImin), CKI, ARTh, and CBI. BAD-D demonstrated the highest AUC of 0.799, followed by ARTmax (0.798), D_TCP-Apex_ (0.771), TBI (0.760), PPImax (0.756), DP_TCP-Apex_ (0.753), and B_Ecc (0.707). We found no significant differences among the AUCs of these 7 metrics by Delong test (all *P* > 0.05). All other analysed indices had an AUC of less than 0.7. No statistically difference could be demonstrated when comparing AUCs of D_TCP-Apex_ and DP_TCP-Apex_ with ARTave AUC (*P*=0.260 and *P*=0.361, respectively). However, the AUCs of D_TCP-Apex_ and DP_TCP-Apex_ were significantly higher than ARTh AUC (*P*=0.004 and *P*=0.008, respectively). The parameters PPImax, D_TCP-Apex_, BAD-D, and PPImin all had 63.64% sensitivity in differentiating VAE-NTB from normal eyes and showed specificities of 88.57%, 87.14%, 85.71%, and 67.14%, respectively.

**Figure 1:**
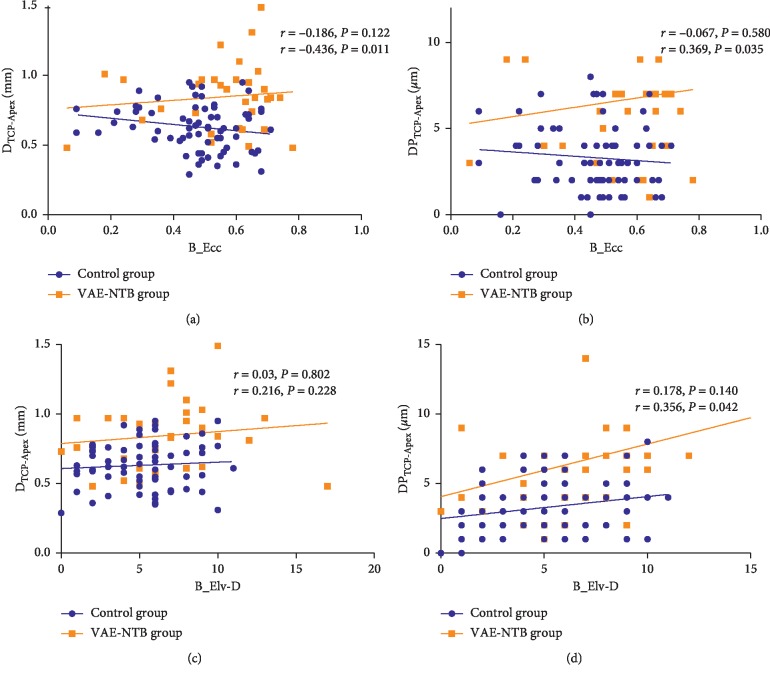
The correlation analysis between the pachymetry distributions (D_TCP-Apex_ and DP_TCP-Apex_) and either B_Ecc or B_Elv-D for the two study groups. (a) The parameter D_TCP-Apex_ is significantly and positively correlated with B_Ecc in the VAE-NTB but not in the control group. (b) The index DP_TCP-Apex_ is significantly and positively correlated with B_Ecc in the VAE-NTB group but not in the control group. (c) The parameter D_TCP-Apex_ has no significant correlation with B_Elv-D in both groups. (d) The parameter DP_TCP-Apex_ is significantly and positively correlated with B_Elv-D in the VAE-NTB but not in the control group.

The relationship between the central pachymetry distributions (both D_TCP-Apex_ and DP_TCP-Apex_) and B_Ecc, as well as B_Elv-D, was analysed separately in the two studied groups (Figure 1). Pachymetry distributions in the control group had no significant correlation with either B_Ecc or B_Elv-D. In the VAE-NTB group, B_Ecc was significantly and positively correlated with D_TCP-Apex_ (*r* = 0.436, *P*=0.011) and DP_TCP-Apex_ (*r* = 0.369, *P*=0.035), whereas B_Elv-D demonstrated a significant positive correlation with only DP_TCP-Apex_ (*r* = 0.356, *P*=0.042), but not with D_TCP-Apex_.

**Figure 2:**
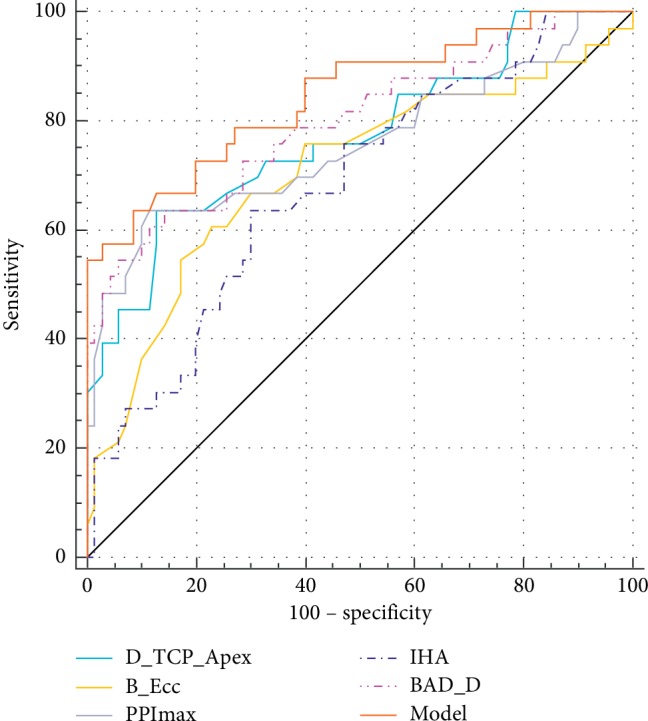
The combined ROC curves for D_TCP-Apex_, B_Ecc, PPImax, IHA, BAD-D, and the combined model using D_TCP-Apex_, B_Ecc, PPImax, and IHA to differentiate VAE-NTB from normal eyes. Note that the model has the highest AUC.

Furthermore, we conducted a multivariable analysis using the significant variables of this study. The result of optimal multiple linear regression model is presented in Table 2. When using the indices D_TCP-Apex_, B_Ecc, PPImax, and IHA, the highest AUC attained was 0.846 revealing a sensitivity of 63.64% and a specificity of 91.43% for detecting VAE-NTB eyes. The ROC curves of this model, D_TCP-Apex_, B_Ecc, PPImax, IHA, and BAD-D are shown in Figure 2. No statistically significant differences were noted in the pairwise comparisons of AUCs between the established regression model and BAD-D, D_TCP-Apex_, and PPImax (all *P* > 0.5), whereas this model had a statistically higher AUC value than B_Ecc and IHA (all *P*=0.009).

## 5. Discussion

Due to the prevailing uncertainty regarding genetic predisposition factors, there remains a gap in detecting the potential patient by molecular diagnostic [21, 22]; thus, screening for subclinical KC at a very early stage remains challenging. To investigate structural alterations of the central cornea at the very early stage of KC, we enrolled study subjects with subclinical KC at a biomechanically compensated stage. We used the relative location distance and pachymetry difference between the corneal thinnest point and the apex to describe pachymetry distribution of the central cornea and evaluate central corneal ectasia in both normal eyes and VAE-NTB individuals. The results showed that the parameters D_TCP-Apex_ and DP_TCP-Apex_ were significantly increased in VAE-NTB than those in the control population, which demonstrated that early ectasia and reduced volume of the central cornea can be detected by Pentacam imaging in subclinical KC with normal biomechanics. Additionally, significant differences were identified in other Scheimpflug indices which referred to corneal pachymetry progression, back elevation, anterior topometric indices, and the parameter BAD-D. The outcomes of the present study demonstrated that focal abnormalities had already occurred before biomechanical failures and topographic abnormalities can be detected. This supported previous hypotheses regarding KC development that progressive thinning and hyperelastic weakening of a cornea produced manifest signs of subclinical KC, even during the period of biomechanical compensation [12, 13].

Several studies reported that the posterior corneal elevation had a relatively good discriminatory ability for subclinical KC screening and that the posterior corneal elevation difference (B_Elv-D) determined with enhanced best fit sphere was better than the posterior corneal elevation alone to diagnose subclinical KC [3, 23, 24]. Eccentricity is a corneal shape factor calculated within a central diameter of 8 mm averaged over all meridians of the corneal surface, to determine a prolate shape or an oblate shape of the corneal surface. Here, we found that the parameters B_Elv-D and B_Ecc were significantly and positively correlated with the index DP_TCP-Apex_ in the VAE-NTB, but not in the control group. Furthermore, a significant positive correlation between D_TCP-Apex_ and B_Ecc was identified in the VAE-NTB group, but not in the control group. These results indicated that the focal corneal thinning and abnormal pachymetry distribution may be responsible for the posterior shape changes at a very early stage of KC. Additionally, those very early posterior abnormalities preceded the changes in curvature and astigmatism in the current study. Our outcomes implied that the metrics regarding the central pachymetry distribution and posterior corneal shape may constitute the most promising variable set for the detection of early ectasia, although the first detectable sign of subclinical KC continues to be controversial.

Ambrósio et al. [7] reported that, to distinguish KC from normal eyes, the pachymetric difference and distance between the thinnest and central points showed AUCs of 0.921 and 0.718, respectively. In the present study, the ROC analysis showed that metrics with AUCs of >0.7 included BAD-D (0.799), ARTmax (0.798), D_TCP-Apex_ (0.771), TBI (0.760), PPImax (0.756), DP_TCP-Apex_ (0.753), and B_Ecc (0.707). The stringent inclusion criteria for the study group may be responsible for the relatively lower AUC, sensitivity, and specificity values when discriminating VAE-NTB from normal eyes. Previously, the established predictors that performed well in discriminating KC patients from normal individuals always showed decreased discriminative power when comparing normal and subclinical KC individuals [15, 25]. The multimetric BAD-D index combined keratometry, pachymetry, pachymetry progression, and back elevation parameters and was considered the best tomographic metric for KC or subclinical KC screening [24, 26, 27]. Ambrósio et al. [25] reported for BAD-D an AUC of 0.997 with 98.2% sensitivity and 99.2% specificity in differentiating KC from normal eyes; however, when comparing the normal and subclinical KC eyes, the BAD-D AUC dropped to 0.838 with decreased sensitivity and specificity of 80.9% and 71.7%, respectively. The parameter PPImax, a competitive predictor for ectatic corneal disease evaluating the spatial distribution of the thickness, has reported an AUC of 0.966 for detecting KC eyes whereas a lower AUC of 0.679 for detecting subclinical KC eyes in the study by Muftuoglu et al. [24].

In the present study, the pairwise comparisons of AUCs with values above 0.7 demonstrated no significant differences among the analysed metrics. Steinberg et al. [15] reported that the AUC values of the indices BAD-D and CBI were similar when comparing subclinical KC and normal eyes (0.784 and 0.787, respectively; *P*=0.484); however, BAD-D demonstrated a relatively higher specificity (79% versus 69%, respectively). We preselected subclinical KC with normal biomechanics as defined by the CBI to compare with normal eyes; therefore, we did not compare the AUC differences between CBI and other analysed parameters. The individual metrics D_TCP-Apex_ and DP_TCP-Apex_ presented accuracy values comparable to those of the complex BAD-D algorithm implying that the central pachymetry distribution is an important factor in the evaluation of early ectasia. This may raise the hope for improvements in the current VAE-NTB screening.

Accumulating evidence demonstrated that the overlap of predictive metrics between healthy and subclinical KC limits early KC detection, and individual metrics poorly distinguish subclinical KC from healthy eyes [15, 24]. The logistic regression analysis of the current study showed that the model combining Scheimpflug imaging variables achieved a fairly good diagnostic level with a higher AUC value of 0.846 than the other analysed metrics. This combined model also had a higher specificity (91.43%) than BAD-D (85.71%), albeit without a statistically significant difference in the pairwise AUC comparison. Several current studies have highlighted the contribution of combined models or algorithms to detect subclinical KC; however, the logistic regression analyses in our study provided only limited improvement in the VAE-NTB screening. Regarding this point, we should take into account the fact that VAE-NTB individuals have a higher level of parametric overlap with normal populations than subclinical KC cases.

There are limitations to this study. First, the current study was limited by its small sample size in the VAE-NTB group due to strict inclusion criteria. Second, the analysed metrics yielded limited sensitivity values of 69.70% or less, but the main analysed metrics (i.e., DP_TCP-Apex_, D_TCP-Apex_, PPImax, and BAD-D) revealed high specificity values of more than 80%. Steinberg et al. [15] reported 69% sensitivity and 79% specificity for BAD-D when comparing normal eyes and subclinical KC with normal topography, whereas the study by Muftuoglu et al. [24] described 60% sensitivity and 90% specificity for normal eyes versus subclinical KC. These low sensitivity values of the current strategies might imply an increased possibility of false-negative results, particularly in the subclinical KC at a very early stage. This is an urgent concern in refractive surgery screening. In future studies, it would be important to perform comprehensive analyses regarding corneal morphology, biomechanics, epidemiology, genetics, and environmental risks to develop strategies with the best discriminatory power for very early subclinical KC eyes. However, future studies with larger sample sizes are warranted to confirm the findings of our study and improve the current VAE-NTB screening.

This study is the first to investigate pachymetry distribution in the central corneal area of VAE-NTB eyes. Our results highlight that central corneal abnormalities including corneal thinning, abnormal pachymetry distribution, and subtle morphologic changes precede detectable biomechanical abnormalities. The combined analysis of the central corneal thinning, location of the corneal thinnest point, and the corresponding corneal surface asymmetry may help to improve the detection of VAE-NTB eyes. However, the studied metrics and predictive model had limited sensitivity to differentiate VAE-NTB from normal eyes. In summary, the identification of very early abnormalities in the central cornea at the clinical disease onset may promote further insight into the development of KC.

## Figures and Tables

**Table 1:** Variables used to differentiate the two study populations.

Parameters	Normal	VAE-NTB	*P* value	AUC	95% CI	Cutoff	Sensitivity (%)	Specificity (%)
fKm (D)^*∗*^	42.66 ± 1.32	42.93 ± 1.22	0.331^‡^	0.52	0.419–0.619	41.4	90.91	21.43
F_astig (D)^*∗*^	1.00 ± 0.53	1.20 ± 0.68	0.138^‡^	0.581	0.480–0.678	1.40	39.39	84.29
F_Ecc^†^	0.54 (0.43, 0.59)	0.60 (0.46, 0.66)	0.075^§^	0.609	0.508–0.704	0.59	51.50	77.10
bKm (D)^†^	−6.25 (−6.40, −6.10)	−6.20 (−6.20, −6.10)	0.119^§^	0.594	0.493–0.690	−6.30	78.79	50.00
B_astig (D)^†^	3.00 (3.00, 4.00)	4.00 (2.00, 5.00)	0.145^§^	0.587	0.486–0.683	0.40	33.33	90.00
B_Ecc^†^	0.49 (0.43, 0.56)	0.61 (0.51, 0.67)	0.001^§^	0.707	0.609–0.792	0.56	60.60	77.10
Kmax (D)^†^	43.90 (43.00, 44.50)	44.2 (42.95, 45.25)	0.199^§^	0.579	0.477–0.675	45.10	27.27	90.00
D_TCP-Apex_ (mm)^*∗*^	0.63 ± 0.16	0.85 ± 0.24	<0.001^‡^	0.771	0.677–0.848	0.79	63.64	87.14
DP_TCP-Apex_ (*μ*m)^†^	3.00 (2.00, 4.00)	6.00 (3.50, 8.00)	<0.001^§^	0.753	0.659–0.833	5.00	57.58	85.71
PPImin^*∗*^	0.73 ± 0.11	0.74 ± 0.16	0.660^‡^	0.519	0.418–0.618	0.68	42.42	72.86
PPImean^*∗*^	1.00 ± 0.10	1.06 ± 0.15	0.039^‡^	0.625	0.524–0.719	1.03	63.64	67.14
PPImax^*∗*^	1.23 ± 0.13	1.42 ± 0.23	<0.001^‡^	0.756	0.662–0.835	1.34	63.64	88.57
ARTave^*∗*^	556.0 ± 73.6	504.0 ± 74.1	0.001^‡^	0.698	0.584–0.813	485.0	48.48	88.57
ARTmax^†^	440.5 (410.0, 479.8)	377.0 (330.0, 426.0)	<0.001^§^	0.798	0.707–0.871	377	51.52	97.14
ISV^*∗*^	14.79 ± 4.26	18.03 ± 4.91	0.001^‡^	0.696	0.598–0.783	18.00	51.52	84.29
IVA^†^	0.10 (0.06, 0.13)	0.14 (0.09, 0.16)	0.003^§^	0.682	0.583–0.771	0.11	66.67	67.14
IHA^†^	4.35 (1.88, 8.40)	8.20 (4.00, 12.20)	0.003^§^	0.681	0.582–0.769	6.40	63.64	70.00
IHD^†^	0.009 (0.005, 0.013)	0.012 (0.008, 0.017)	0.032^§^	0.631	0.530–0.724	0.013	42.42	85.71
KI^†^	1.02 (1.00, 1.03)	1.03 (1.02, 1.05)	0.007^§^	0.665	0.565–0.755	1.02	57.58	67.14
CKI^†^	1.01 (1.00, 1.01)	1.01 (1.00, 1.01)	0.659^§^	0.524	0.423–0.623	1.01	9.09	98.57
B_Elv-D^†^	5.00 (3.00, 7.00)	7.00 (4.50, 9.00)	0.012^§^	0.653	0.552–0.744	6.00	57.58	74.29
BAD-D^*∗*^	0.79 ± 0.4	1.5 ± 0.7	<0.001^‡^	0.799	0.708–0.871	1.20	63.64	85.71
ARTh^†^	468.8 (425.2, 510.7)	479 (422.4, 530.1)	0.398	0.552	0.451–0.650	512.1	39.39	78.57
CBI^†^	0.01 (0.00, 0.04)	0.02 (0.00, 0.09)	0.074^§^	0.606	0.505–0.701	0.04	42.42	80.00
TBI^†^	0.07 (0.01, 0.18)	0.27 (0.09, 0.74)	<0.001^§^	0.760	0.666–0.838	0.17	69.70	75.71

fKm, front mean keratometry; F_astig, front astigmatism; F_Ecc, front eccentricity; bKm, back mean keratometry; B_astig, back astigmatism; B_Ecc, back eccentricity; Kmax, maximum keratometry; DTCP-Apex, distance between the corneal thinnest point and the apex; DPTCP-Apex, pachymetry difference between the corneal thinnest point and the apex; PPImin, PPImean, and PPImax, minimum, mean, and maximum pachymetry progression index, respectively; ARTave and ARTmax, average and maximum Ambrósio's relational thickness; ISV, index of surface variation; IVA, index of vertical asymmetry; IHA, index of height asymmetry; IHD, index height decentration; KI, keratoconus index; CKI, central keratoconus index; B_Elv-D, posterior corneal elevation difference; BAD-D, Belin-Ambrósio deviation; ARTh, Ambrósio's relational thickness to the horizontal profile; CBI, Corvis Biomechanical Index; TBI, tomography and biomechanical index; Normal, normal eyes; VAE-NTB, forme fruste keratoconus with normal biomechanics; AUC, area under the curve; CI, confidence interval. ^*∗*^Values are listed as the mean ± standard deviation. ^†^Values are listed as median (M25, M75). ^‡^Independent *t*-test. ^§^Mann–Whitney *U* test.

**Table 2:** Stepwise multiple linear regression model analysis for the predictive model.

Main predictors	B	SE	Wald	*P* value
Constant	−13.401	3.072	19.025	<0.001
D_TCP-Apex_	3.683	1.728	4.540	0.033
B_Ecc	4.804	1.997	5.786	0.016
PPImax	5.067	2.186	5.372	0.02
IHA	0.126	0.056	5.079	0.024

B, unstandardized coefficients; SE, standard error of B; D_TCP-Apex_, distance between the corneal thinnest point and the apex; B_Ecc, back eccentricity; PPImax, maximum pachymetry progression index; IHA, index of height asymmetry.

## Data Availability

The datasets used and/or analysed during the present study are available from the corresponding author on reasonable request.
